# Improvement of Depression after Treatment of Dural Arteriovenous Fistula: A Case Report and a Review

**DOI:** 10.1155/2012/730151

**Published:** 2012-11-28

**Authors:** Minoru Nakagawa, Kenji Sugiu, Koji Tokunaga, Chihoko Sakamoto, Kenjiro Fujiwara

**Affiliations:** ^1^Department of Neurosurgery, Kosei General Hospital, 2-5-1 Enichi-cho, Mihara 7238686, Japan; ^2^Department of Neurological Surgery, Okayama University Graduate School of Medicine, Dentistry and Pharmaceutical Sciences, Okayama 700-8558, Japan; ^3^Department of Neurology, Kosei General Hospital, Mihara 7238686, Japan

## Abstract

Patients with dural arteriovenous fistulas (DAVFs) in the transverse-sigmoid sinus suffer from several symptoms: bruit, headache, visual impairment, and so on. But depression is rare in patients with DAVF. The authors reported a rare case presenting the improvement of depression after the treatment of a dural arteriovenous fistula in the left transverse-sigmoid sinus. A 46-year-old male had suffered from depression and was treated with antidepressants at a local hospital for four years. The patient was temporarily laid off due to his depression. Afterwards, he had Gerstmann's syndrome and came to our hospital. A DAVF in the left transverse-sigmoid sinus was demonstrated on the angiogram. The DAVF was successfully treated with endovascular surgery, coil embolization of the isolated diseased sinus through the mastoid emissary vein which was a draining vein from the fistula. After this treatment, his depression as well as Gerstmann's syndrome was improved and the quantity of the antidepressants decreased. The patient returned to work without any antidepressant two years after the treatment. DAVFs might be one of the causes of depression. It may be necessary to evaluate cerebral vessels in patients suffering from depression by using MRA or 3D-CTA even if there are not any abnormal findings on plain CT scans.

## 1. Introduction

Dural arteriovenous fistulas (DAVFs) are thought to be acquired lesions, resulting from collateral revascularization following thrombosis of a venous sinus [1]. Patients with DAVFs in the transverse-sigmoid sinus suffer from several symptoms: bruit, headache, visual impairment, and so on. But depression is very rare in patients with DAVF. We present a patient with a DAVF in the transverse-sigmoid sinus that was thought to cause depression and discuss the mechanism of depression occurred by a DAVF.

## 2. Case Presentation

**Figure 1:**
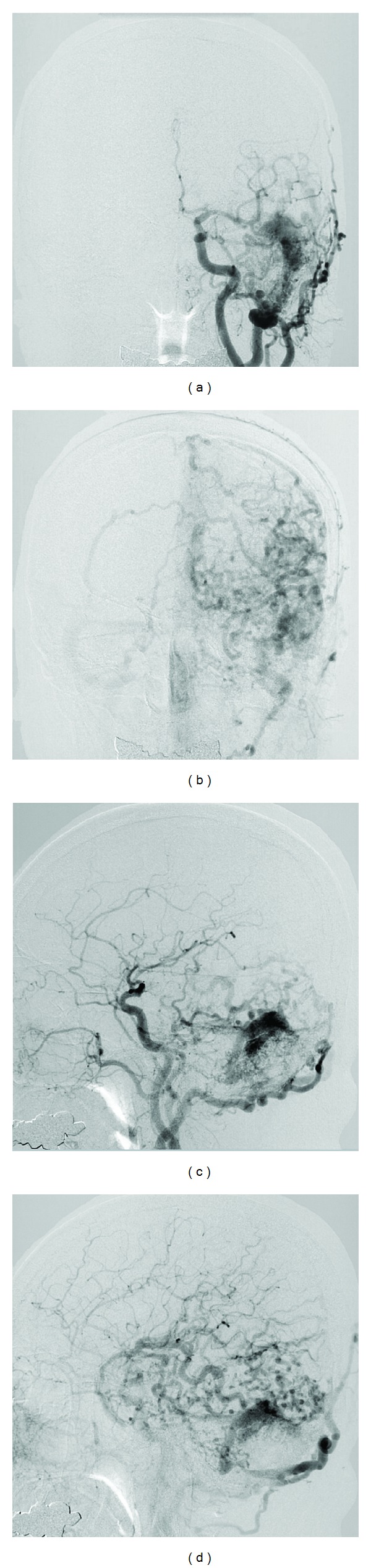
(a) A DAVF in the left transverse-sigmoid sinus was shown on the arterial phase of the left common carotid artery angiogram (anteroposterior view). (b) Numerous draining cortical veins of the AVF indicating severe venous reflux and congestion were demonstrated on the venous phase of the angiogram (anteroposterior view). (c) On the arterial phase of the lateral view, a DAVF had many feeding arteries, meningeal branches from the external carotid artery, cortical venous reflux, and the isolated left transverse-sigmoid sinus as drainer [2, 3]. (d) Note the cortical venous congestion in the wide area of the left cerebral hemisphere [2, 3].

**Figure 2:**
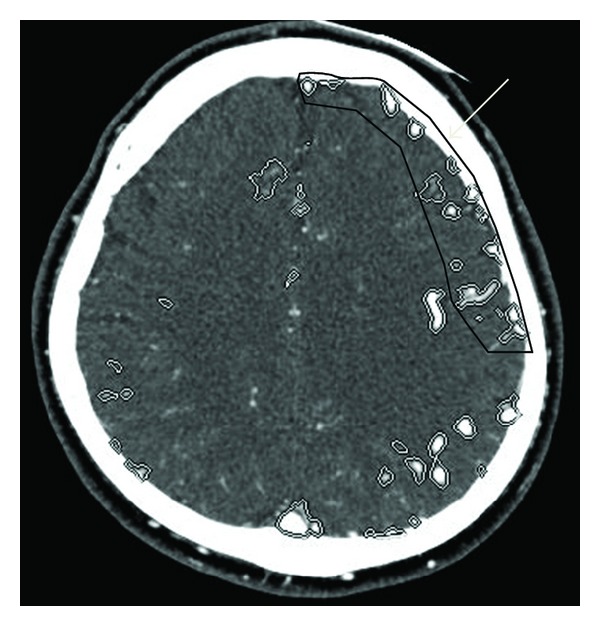
In a former axial image of 3D-CT angiography arterial phase obtained after admission, abnormal vessels (circle markings) appeared in the left cerebral hemisphere including the DLPFC (arrow).

**Figure 3:**
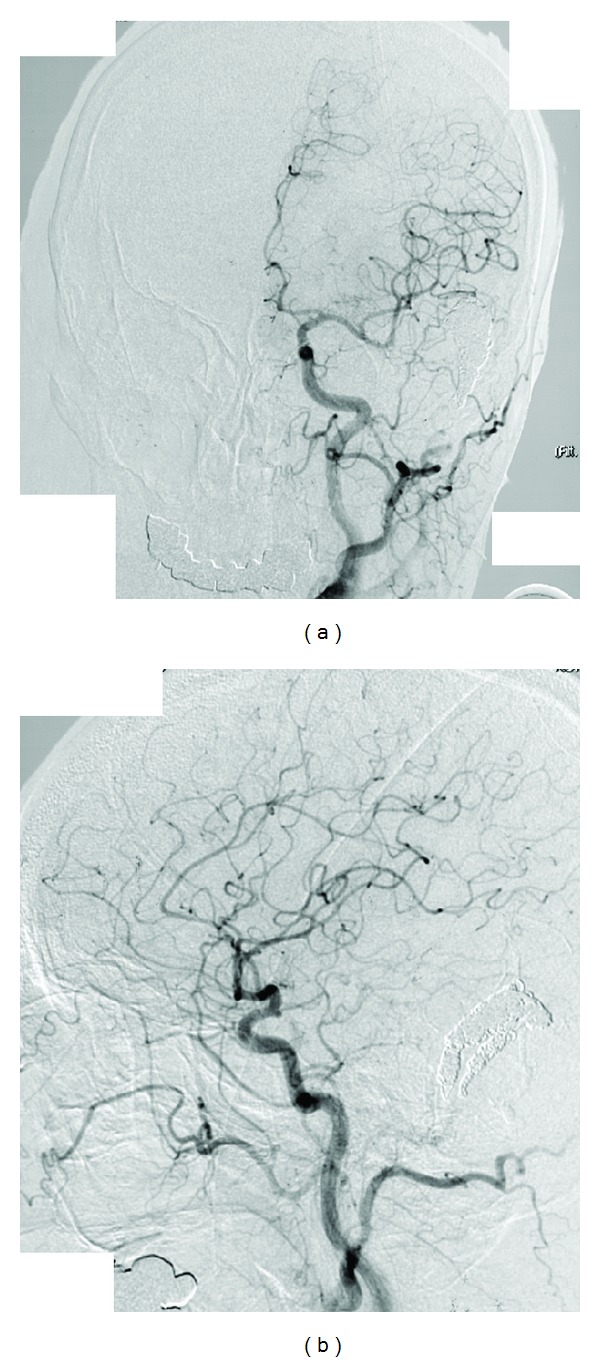
A DAVF disappeared on the angiograms (a) anteroposterior view and (b) lateral view [2, 3].

Part of this case has already been reported previously [2–4]. A 46-year-old male had been treated with antidepressants to depression at a local hospital for four years. The patient was temporarily laid off due to his depression. Afterwards, he became poor at ordinary conversation with his wife. Therefore, she took him to the local hospital. Because a cerebrovascular disease was suspected in the institute and the patient was recommended to consult neurosurgery, he came to our hospital on the same day as onset of the symptoms. On admission, the patient exhibited acalculia, finger agnosia, right-left disorientation, agraphia, and hemispatial agnosia. A CT scan was obtained immediately. Multiple high density spots were evident in a wide area of the left temporal, parietal, and occipital lobes on plain CT scan. They were thought to be dilated draining cortical veins. The left transverse-sigmoid sinus corresponding to the draining sinus also became larger than the right side on the CT scan [2]. A cerebral angiography was subsequently performed. A DAVF in the left transverse-sigmoid sinus with cortical vein reflux in the left temporal, parietal, and occipital lobes was demonstrated on the left common carotid artery angiogram. Its feeding arteries were the left occipital artery, posterior auricular artery, middle meningeal artery, and superficial temporal artery (Figure 1). After the examinations, the patient was admitted to the intensive care unit. In a former axial image of 3D-CT angiography arterial phase obtained after admission, abnormal vessels appeared in the left cerebral hemisphere including the left dorsolateral prefrontal cortex (DLPFC) (Figure 2). After the three endovascular surgeries [4], the DAVF including the cortical vein reflux disappeared on the angiogram (Figure 3). The patient did not experience any complications during and after these procedures, and his depression as well as Gerstmann's syndrome was improved. The quantity of the antidepressants for the patient decreased after the treatment, and the patient returned to work without any antidepressant two years after the treatment.

## 3. Discussion

Patients with DAVFs in the transverse-sigmoid sinus suffer from several symptoms: bruit, headache, visual impairment, and so on. But depression is very rare in patients with DAVF. Katz et al. reported the first case suffering from reversible major depression due to a giant sinus dural AV fistula [5]. After the endovascular embolization, a brain SPECT scan demonstrated enhanced perfusion of the left frontoparietal lobe, especially the left DLPFC, and the depression was improved. In the present case, wide-spreading abnormal vessels appeared in the left DLPFC on the arterial phase of CT angiography. And a cortical venous reflux disappeared on the angiogram after the transvenous embolization with coil for DAVF. Therefore, the improvement of cerebral blood flow in the left DLPFC was thought to be obtained. Pizzagalli et al. pointed out the importance of a left DLPFC in cases with major depressive disorder. Decreased functional activity of the left DLPFC is most frequently implicated on EEG, SPECT, and PET scans in their report [6]. Moreover, the function of the left dorsolateral and left medial prefrontal cortex where the expectancy of pleasant stimuli produces activation decreased in patients with depression on the functional MRI study [7]. In another functional MRI study, the DLPFC, inferior parietal cortex, dorsal raphe nucleus, and cerebellum are activated when subjects learn to act in order to obtain large future rewards related with serotonin which decreases in patients with depression. Impairment of the activity on future reward prediction is thought to make patients despondent, pessimistic, and apathic [8]. The DAVF might impair the functional activity of these areas also in our case. The cause of transient Gerstmann's syndrome in our patient is unclear. Katz et al. considered the cause of the specific precipitant of an acute global aphasia in their patient, that is, a sudden shift of DAVF drainage following a new focal thrombosis may have altered left hemispheric outflow [5]. As another possibility, an epilepsy might be occurred by venous congestion due to DAVF. Our patient is the rare case of a DAVF presenting with the sudden onset of transient neurological deficit in a patient with depression, whose deficits resolved after therapeutic obliteration, and following the case reported by Katz et al [5]. DAVFs might be one of the causes of depression. It may be necessary to evaluate cerebral vessels in patients suffering from depression by using MRA or 3D-CTA even if there are not any abnormal findings on plain CT scans in DAVFs.
